# Supplementary material for: An update on Scaponopselaphus Scheerpeltz (Coleoptera: Staphylinidae) with the description of three new species, and a key to the species
Source: PLoS One. 2025 Jul 23;20(7):e0326484. doi: 10.1371/journal.pone.0326484 (PMC12286392; doi:10.1371/journal.pone.0326484)
Supplement: Attachment — Submitted filename: rev.15.03.2025Scaponopselaphus_final2.docx [file pone.0326484.s005.docx]

An update on *Scaponopselaphus* Scheerpeltz (Coleoptera: Staphylinidae) with the description of three new species, a key to the species, and its ecological niche modeling in South America

Louri Klemann-Junior^1,2*^, Angélico Asenjo^3,4*^, Bruno Gouvea^5^, Roberta M. Valente^5^

^1^ Laboratório de Biodiversidade, Ecologia e Conservação de Ecossistemas Amazônicos, Universidade do Estado do Amazonas, Centro de Estudos Superiores de Itacoatiara, Itacoatiara, Amazonas, Brazil

^2^ Programa de Pós-Graduação em Ciência e Tecnologia para Recursos Amazônicos, Instituto de Ciências Exatas e Tecnologia, Universidade Federal do Amazonas, Itacoatiara, Amazonas, Brazil

^3^ Facultad de Ciencias Biológicas, Universidad Nacional Mayor de San Marcos, Lima, Peru

^4^ Departamento de Entomología, Museo de Historia Natural de la Universidad Nacional Mayor de San Marcos, Peru

^5^ Laboratório de Invertebrados (LA-INV), Instituto de Ciências Biológicas, Universidade Federal do Pará, Belém, Pará, Brazil

* Corresponding author

Email: klemannjr@yahoo.com.br (LKJ); angelico.asenjo@unmsm.edu.pe (AA)

All authors contributed equally to this work.

# **Abstract**

Three new species of the genus *Scaponopselaphus* Scheerpeltz, 1972 are described; *Scaponopselaphus caribi* **sp. nov.**, and *Scaponopselaphus oby* **sp. nov.** from Brazil, and *Scaponopselaphus paradoxus* **sp. nov.** from Peru. New locality for *Scaponopselaphus mutator* (Sharp, 1876) is recorded. Major diagnostic features and a pictorial and dichotomic identification key are provided for all species of the genus. Comments about the current and potential distribution by the niche modeling analysis of the genus *Scaponopselaphus* in South America are also provided.

# **Introduction**

*Scaponopselaphus* Scheerpeltz is a genus of rove beetles distributed in the Amazon Rainforest (see Guayasamin et al. [1] for biome limits), with occurrence in Peru, Colombia, Guiana, Suriname, French Guiana, and Brazil at altitudes ranging from 30 to 317 m [2, 3]. The genus, up to now, accommodates only two species and was revised by Chatzimanolis [2]. This genus is rarely collected and poorly represented in entomological collections. Chatzimanolis in his revision redescribed the type species of the genus, *Trigonopselaphus mutator* Sharp 1876, described *Scaponopselaphus diaspartos* Chatzimanolis 2015 from Colombia, and provided distribution maps and illustrations of structural features.

Despite its wide distribution across several countries, *Scaponopselaphus* Scheerpeltz has scattered occurrences and is often separated by large gaps, such as the central region of the Amazon biome, suggesting under-sampling and/or occupation of a specific ecological niche. The limited presence of this taxon in collections highlights the challenge of understanding the true diversity of the genus. To address these challenges, ecological niche modeling has emerged as a powerful tool to predict potential distribution areas based on known occurrence records and environmental variables [4], helping to guide future sampling efforts, and refine our understanding of the genus.

In this article, we describe three new species of *Scaponopselaphus* Scheerpeltz, two from Brazil and one species from Peru, providing photographs, illustrations and measurements. We also provide a pictorial and dichotomic identification key to all species of the genus. Finally, we analyze the geographic distribution of *Scaponopselaphus* and furnish the first ecological niche modeling analysis for the genus.

# **Material and methods**

## **Morphological analyses**

*Specimens*. All male specimen studied here were relaxed in warm soapy water and the apical abdominal segments containing the aedeagus were dissected from the abdomen. First, the apical abdominal segments were cleared using 10% KOH. Then, the aedeagus was removed from the inside of the abdomen. The dissections and characters studies were carried out under a stereoscopic microscope. The photographs of *Scaponopselaphus caribi* **sp. nov.** were taken using a Canon 5D mark IV digital camera equipped with an extension tube and a MP-E65 mm macro lens. The photographs of *Scaponopselaphus oby* **sp. nov.** and *Scaponopselaphus paradoxus* **sp. nov.** were taken using a stereo microscope Leica M205A equipped with a Leica DFC420 digital camera. Photographs of the aedeagus of *Scaponopselaphus caribi* **sp. nov.** were taken using a five-megapixel camera attached to a trinocular optical microscope. The images were automontaged on Helicon Focus 8 software. The adopted terminology for the descriptions follows Naomi [5, 6] and Chatzimanolis [2].

## **Characters and measurements**

The description and measurements of the new species are based on the holotype, additional characters are included in the remarks section. In addition to the data previously described for the holotypes, we provided measurements (Fig 1) for all studied specimens (S1 Table) and the range of measurements for each structure, with the average in parentheses in the measurement sections. All measurements are in millimeters and were taken under a Leica M205A (7.8X–128X) stereomicroscope (Fig 1).

**Fig 1. *Scaponopselaphus* spp. Measurements taken**. **Body:** BL = body length, BW = maximum width of the elytra; **Head:** HL = maximum length of the head, HW= maximum width of the head including eyes, NW = maximum width of the neck, OL = maximum length of the eyes in dorsal view, OW = maximum width of the eyes in dorsal view, IO = minimum interocular distance; **Antenna:** AL = antennomere length; AW = antennomere width; **Thorax:** PL = pronotal length along the midline, PW = maximum width of the pronotum, EL = elytral length, EW = maximum width of the elytra, SL = scutellar shield length, SW = scutellar shield width.

In the type label data, quotation marks (“”) separate different labels, and a vertical bar (|) separates different lines within a label. Text within square brackets [ ] is explanatory and it is not included in the original labels.

## **Study area and occurrence records**

The current study focuses on the South American continent, with 55 occurrence records compiled from: Chatzimanolis [2], specimen labels studied in this publication, and records from the Global Biodiversity Information Facility (GBIF) [7]. The current distribution map (Fig 2) was produced using QGIS 3.38 software (under the GNU General Public License) [8] and a KMZ map for Google Earth in S1 Map.

**Fig 2. Current distribution of *Scaponopselaphus* Scheerpeltz**.

## **Distribution analysis**

For occurrence filtering, the occurrence data were thinned based on a distance of twice the cell size. For pseudo-absence allocation, we used the Environmental Constrain method, which restricts areas with the lowest suitability, employing a presence-absence ratio of 1. For climate data, we utilized the WorldClim v2.1 database for 1970–2000 [9]; (found at http://www.worldclim.org), at a resolution of 30 arc seconds (1 km²). Initially, 19 bioclimatic variables from 1970 to 2000 were selected to generate the models: Annual Mean Temperature (BIO1), Mean Diurnal Range (BIO2), Isothermality (BIO3), Temperature Seasonality (BIO4), Max Temperature of Warmest Month (BIO5), Min Temperature of Coldest Month (BIO6), Temperature Annual Range (BIO7), Mean Temperature of Wettest Quarter (BIO8), Mean Temperature of Driest Quarter (BIO9), Mean Temperature of Warmest Quarter (BIO10), Mean Temperature of Coldest Quarter (BIO11), Annual Precipitation (BIO12), Precipitation of Wettest Month (BIO13), Precipitation of Driest Month (BIO14), Precipitation Seasonality (BIO15), Precipitation of Wettest Quarter (BIO16), Precipitation of Driest Quarter (BIO17), Precipitation of Warmest Quarter (BIO18), and Precipitation of Coldest Quarter (BIO19). We applied a Principal Component Analysis (PCA) to reduce collinearity and used the first six axes as new predictors [10]. For modeling, we have used four algorithms: Maximum Entropy default “MAXENT” [11, 12], Support Vector Machine “SVM” [13]; Generalized Linear Model “GLM” ([R Core Team, 2018)](https://www.sciencedirect.com/science/article/pii/S1364815219310424#bib72) [14]; Random Forest “RDF” [15]. For model validation, we applied the Bootstrap partition method [16] with 20 replicates, using 70% of the data for training and 30% for testing across the entire dataset. Considering the sensitivity of True Skill Statistics “TSS” and Area Under the Curve “AUC” to species prevalence [17], the threshold for presence-absence predictions was determined as the value at which Jaccard index was the highest, and the model's accuracy was estimated with the metrics of “AUC” [16], “TSS” [18], and Jaccard [17]. An ensemble model was created by calculating the simple average of the predictions of different models. All models, metrics, and methods used here were processed with the “ENMTML” package [19] in R 4.4.2 environment [20]. The maps were produced using QGIS 3.38 [7].

## **Depositaries**

The specimens studied are deposited in the following collections (curator(s) in parentheses).

**INPA:** Instituto Nacional de Pesquisas da Amazônia, Manaus, Amazonas, Brasil (Dr. Márcio Luiz de Oliveira).

**MPEG:** Museu Paraense Emilio Goeldi, Belém, Pará, Brazil (Dr. Orlando Tobias Silveira).

**MUSM:** Museo de Historia Natural, Universidad Nacional Mayor de San Marcos, Lima, Lima, Peru (Dra. Diana Silva & Dr. Gerardo Lamas).

**UEA:** Entomological Collection of Centro de Estudos Superiores de Itacoatiara, Universidade do Estado do Amazonas, Itacoatiara, Amazonas, Brasil (Dr. Louri Kleman-Junior).

**UFPA:** Collection of Coleoptera of the Universidade Federal do Pará (Dra. Roberta Valente).

# **Nomenclatural acts**

The electronic edition of this article conforms to the requirements of the amended International Code of Zoological Nomenclature, and hence the new names contained herein are available under that Code from the electronic edition of this article. This published work and the nomenclatural acts it contains have been registered in ZooBank, the online registration system for the ICZN. The ZooBank LSIDs (Life Science Identifiers) can be resolved and the associated information viewed through any standard web browser by appending the LSID to the prefix “http://zoobank.org/”. The LSID for this publication is: urn:lsid:zoobank.org:pub:C769B82C-66DD-4416-BBDC-CA37DCD944DD. The electronic edition of this work was published in a journal with an ISSN, and has been archived and is available from the following digital repositories: PubMed Central and LOCKSS.

# **Results**

Subfamily Staphylininae Latreille, 1802

Tribe Staphylinini Latreille, 1802

Subtribe Xanthopygina Sharp, 1884

Genus *Scaponopselaphus* Scheerpeltz, 1972

## ***Scaponopselaphus caribi* Klemann-Junior, Asenjo, Gouvea & Valente, sp. nov.** urn:lsid:zoobank.org:act:60504E61-37B9-45BF-AA44-D0F2905263B5

(Figs 2, 3A–C, 4A–G, 5A–F and 12)

**Type material** (2 males, 1 female)

**Holotype.** male: “Brasil: AM[Amazonas state], Itapiranga[municipality] | Faz.[farm] Caribi - Prato rosa[pan trap - pink color] | 28.iv[April]-01.v[May].2022, T2/A1[trap code] | 2°32'50,48''S/58°48'23,16''W | L.Klemann-Junior | CESIT - UEA [left sideline]”, “HOLOTYPE [red label] | *Scaponopselaphus* | *caribi* sp. nov. | Desig. Klemann-Junior *et al*. 2025”, “INPA-COL 002848” (INPA).

**Paratypes.** 1 male, 1 female: “Brasil: AM [Amazonas state], Manaus, Embrapa[Brazilian Agricultural Research Corporation] | Guaraná convencional - mata[Conventional guarana cultivation - forest] | 11.x[October].2012 - Moerick trap | 2°53'42,18''S/59°59'10,58''W | K. Schoeninger” (male, INPA). “Brasil: AM [Amazonas state], Silves | Faz.[farm] Itapiranga | 04-11.v[May].2019 I[rainy season]11/12 [abbreviated logging year]T5A1 [trap code] | 2°41'25,31''S/58°33'42,82''W | L. F. B. Pereira | CESIT - UEA [left sideline]” (female, UEA). All paratypes with label: “PARATYPE [yellow label] | *Scaponopselaphus* | *caribi* sp. nov. | Desig. Klemann-Junior *et al*. 2025”.

**Diagnosis.** Among the known *Scaponopselaphus* species, *Scaponopselaphus caribi* **sp. nov.** is the unique species that has a black elytra (Fig 3) and the apex of the lobes of paramere of aedeagus obliquely truncated (Fig 5).

**Fig 3**. **Holotype of *Scaponopselaphus caribi* sp. nov. (INPA-COL 002848).** **A.** Dorsal habitus; **B.** Head and pronotum; **C.** Antenna.

**Measurements** males [min–max (average); N = 2]: ***Body***. BL = 10.88–11.29 (11.09), BW = 2.72–2.76 (2.74). ***Head***. HL = 1.33–1.35 (1.34), HW = 1.80–1.87 (1.835), NW = 1.01–1.05 (1.03), OL = 0.74–0.85 (0.795), OW = 0.31–0.35 (0.33), IO = 1.18–1.18 (1.18). ***Antenna***. antennomere 1 AL = 0.48–0.49 (0.485), AW = 0.17–0.17 (0.17), antennomere 2 AL = 0.24–0.25 (0.245), AW = 0.15–0.15 (0.15), antennomere 3 AL = 0.29–0.30 (0.295), AW = 0.16–0.16 (0.16), antennomere 4 AL = 0.17–0.20 (0.185), AW = 0.20–0.22 (0.21), antennomere 5 AL = 0.18–0.20 (0.19), AW = 0.22–0.24 (0.23), antennomere 6 AL = 0.17–0.18 (0.175), AW = 0.22–0.24 (0.23), antennomere 7 AL = 0.18–0.18 (0.18), AW = 0.22–0.24 (0.23), antennomere 8 AL = 0.17–0.17 (0.17), AW = 0.21–0.23 (0.22), antennomere 9 AL = 0.17–0.17 (0.17), AW = 0.21–0.22 (0.215), antennomere 10 AL = 0.18–0.18 (0.18), AW = 0.20–0.21 (0.205), antennomere 11 AL = 0.21–0.23 (0.22), AW = 0.18–0.18 (0.18). ***Thorax***. PL = 1.90–2.00 (1.95), PW = 2.07–2.15 (2.11), EL = 2.38–2.42 (2.40), EW = 2.72–2.76 (2.74), SL = 0.51–0.63 (0.57), SW = 0.78–0.84 (0.81).

**Measurements** female [N = 1]: ***Body***. BL = 11.13, BW = 2.67. ***Head***. HL = 1.23, HW = 1.78, NW = 1.03, OL = 0.75, OW = 0.33, IO = 1.13. ***Antenna***. antennomere 1 AL = 0.47, AW = 0.16, antennomere 2 AL = 0.26, AW = 0.14, antennomere 3 AL = 0.28, AW = 0.16, antennomere 4 AL = 0.16, AW = 0.21, antennomere 5 AL = 0.16, AW = 0.22, antennomere 6 AL = 0.16, AW = 0.22, antennomere 7 AL = 0.16, AW = 0.22, antennomere 8 AL = 0.17, AW = 0.23, antennomere 9 AL = 0.17, AW = 0.22, antennomere 10 AL = 0.18, AW = 0.20, antennomere 11 AL = 0.23, AW = 0.18. ***Thorax***. PL = 1.95, PW = 2.13, EL = 2.35, EW = 2.67, SL = 0.57, SW = 0.81.

**Description**. Holotype male, BL: 11.29, BW: 2.76.

**Coloration**. Head and pronotum dark metallic purple-blue (Figs 3A–3B). Antenna (Fig 3C) with antennomere 1 dark reddish brown with lighter apex, antennomere 2 reddish brown with lighter apex, antennomeres 3–10 dark reddish brown, and antennomere 11 dark reddish brown with yellowish apical third. Legs dark reddish brown (Fig 3A) with tarsi becomes lighter from tarsomere 1 to 4. Elytra (Fig 3A) shiny black. Abdomen (Figs 3A and 4A) dark reddish brown, almost black, except posterior third of segment VII and segments VIII–IX reddish brown. Prosternum, meso- and metaventrite dark reddish brown.

**Head** (Figs 3A–3B) wider (HW: 1.87) than long (HL: 1.35) with rounded hind angles, clypeus emarginated, surface of vertex flat. Head with long Dark brown macrosetae along borders of head, with sparse umbilicate punctures each carrying dark brown microsetae, umbilicate punctures absent in middle and denser posteriorly. Integument of vertex with dense microsculpture formed by fingerprint-like microlines uniformly distributed. Anterior margin of frontoclypeal region emarginated (Fig 3B). Labial palpi with apical segment securiform with expanded apex (Figs 4D–4E). Eyes large and prominent (OL: 0.85; OW: 0.35), distance between eyes (IO) 1.18. Antennomeres (Fig 3C) 1–3 longer than wide, gradually club-like thickened, and without microtrichae; antennomere 1 (AL: 0.49; AW: 0.17), antennomere 2 (AL: 0.25; AW: 0.15), antennomere 3 (AL: 0.29; AW: 0.16); antennomeres 4–10 transverse and just slightly asymmetrical, antennomere 4 (AL: 0.17; AW: 0.22), antennomere 5 (AL: 0.18; AW: 0.24) antennomere 6 (AL: 0.17; AW: 0.24), antennomere 7 (AL: 0.18; AW: 0.24), antennomere 8 (AL: 0.17; AW: 0.23), antennomere 9 (AL: 0.17; AW: 0.22), antennomere 10 (AL: 0.18; AW: 0.21); antennomere 11 longer than wide (AL: 0.21; AW: 0.18); antennomeres 4–11 densely covered by microtrichae. Dorsal surface of neck with microsculpture like to vertex of head (Fig 3A–3B).

**Fig 4**. **Holotype of *Scaponopselaphus caribi* sp. nov. (INPA-COL 002848).** **A.** Abdominal sternites V–IX; **B.** Abdominal sternite VIII; **C.** Abdominal sternite IX; **D.** Head, ventral view; **E.** Labial palpomeres, lateral view; **F.** Mesotarsus, lateral view; **G.** Mesotarsus, ventral view.

**Pronotum** (Figs 3A–B) wider than long (PL: 2.00; PW: 2.15), with anterior margin straight, lateral margin concave with hind lateral angles and posterior margin rounded. Disk with dark brown microsetae and, along borders, few dark brown macrosetae. Scattered medium size umbilicate punctures, denser near anterolateral corners; punctures absent along midline and near posterolateral corners. Integument of pronotum shining, with dense microsculpture formed by fingerprint-like microlines uniformly distributed. Scutellum with dense dark brown microsetae and punctures covering its surface, except posterolateral borders.

**Elytra** (Fig 3A) wider than pronotum (EL: 2.42; EW: 2.76); appearing shining; its dorsal surface with denser punctuation than on pronotum with punctures almost confluent, and covered by dark brown setae and, along borders, by few dark brown macrosetae.

**Legs** (Fig 3A) with brown setae; protarsus dorsoventrally flattened and with spatulate pale setae ventrally; mesotarsomere 1 with spatulate pale setae ventrally (Figs 4F–4G). Mesotibia and metatibia with multiple rows of spurs on external side and with apical spurs. Protibia without multiple rows of spurs on external side; with single row of spurs apically.

**Abdomen** (Figs 3A and 4A) with dense and uniform punctuation pattern; punctures absent anteriorly to subbasal carina in segments III–V and anteriorly to basal carina in segment VI; each puncture with dark brown crosetae on segments III–VI and anteriorly to 2/3 of segment VII; yellowish crosetae on posterior third of segment VII and on segment VIII. Tergites and sternites with dense microsculpture formed by mesh-like microlines uniformly distributed. Few long dark brown macrosetae along lateral borders of all segments. Abdominal tergites III–VI with one undulating tergal basal carina; tergites III–V with subbasal one undulated carina; it is shallowest in tergite V. Sternite VII with round porose structure anteriorly (Fig 4A); its posterior margin of sternite VII with shallow and broad emargination at midline (Fig 4A); posterior margin of sternite VIII with deep U-shaped emargination at midline (Fig 4B); sternite IX with shallow U-shaped emargination at midline (Fig 4C),. it is Tergite IX long and straight, and covered with long dark brown setae and with some dark macrosetae (Fig 4A).

**Aedeagus** as in Figures 5A–5F mm long . Paramere in parameral view (Fig 5B) divided longitudinally in two lobes shorter than median lobe (Fig 5A–5B), and converging to obliquely truncated apex (Figs 5B and 5E), this last with peg setae arrangement in at most three rows (Figs 5E–5F). Sides of median lobe in parameral view (Fig 5B) converging to broad pointed apex, and with one small wide bicuspid tooth (Fig 5D),; in lateral view tapering apically (Figs 5A and 5D).

**Fig 5.** **Aedeagus of *Scaponopselaphus caribi* sp. nov. Holotype (INPA-COL 002848)**. **A.** Lateral view; B**.** Parameral view; **C.** Median lobe with paramere removed, parameral view; **D.** Detail of the tip of median lobe, oblique view; **E.** Detail of the tip of paramere, underside; **F.** Detail of the tip of paramere, oblique view.

**Female**. Similar to male but vertex of head is slightly convex; head and pronotum are lighter blue with green overtones, posterior margin of sternites VII and VIII straight, and mesotarsomere 1 without spatulate pale setae ventrally.

**Habitat**. Collected with Pan trap (pink color), with Moerick trap, and with Pitfall trap installed on the ground (baited with fresh human and pig feces 1:9 ratio) (see Moura et al. [21] for details). The capture sites belong to the company Precious Woods – Mil Madeiras Preciosas and are destined for selective logging. The vegetation in the area is Evergreen Tropical Forest “Floresta Ombrófila Densa de Terras Baixas” (Veloso et al. [22]).

**Known distribution** (Fig 2). Know from municipalities of Silves and Itapiranga in the state of Amazonas, Brazil.

**Etymology**. The specific epithet refers to the name of the river (Rio Caribi) near the collection site of the holotype. The name of the river is a reference to indigenous populations of Karib language family who inhabit the frontier region between Brazil (East Paru River, state of Pará), Surinam (Tapanahoni and Paloemeu rivers) and French Guiana (upper Maroni River and its tributaries the Tampok and Marouini). The specific epithet is a noun in apposition.

## ***Scaponopselaphus oby* Asenjo, Gouvea, Valente & Klemann-Junior, sp. nov.** urn:lsid:zoobank.org:act:38675612-4D7F-4F5E-B9AE-85206EF56632

(Figs 2, 6A–C, 7A–H, 8A–F and 12)

**Type material** (2 males, 1 female)

**Holotype.** male: “STP0360 | BRAZIL: Paragominas, | Mineradora Hydro. AL2, | S 03°15'44'' W 047°42'26''. | 05-V[May]-18[2018]. Winkler”, “HOLOTYPE [red label] | *Scaponopselaphus* | *oby* sp. nov. | Desig. Klemann-Junior *et al*. 2025” (MPEG).

**Paratypes.** 1 male, 1 female: “STP0358 | BRAZIL: Paragominas, | Mineradora Hydro. AL9, | S 03°15'43'' W 047°42'07''. | 8-V[May]-18[2018]. p. amarelo[yellow trap]” (1 male, UFPA).

“STP0357 | BRAZIL: Paragominas, | Mineradora Hydro. AL9, | S 03°15'43'' W 047°42'07''. | 8-V[May]-18[2018]. p. amarelo[yellow trap]” (1 female, UFPA).

All paratypes with label: “PARATYPE [yellow label] | *Scaponopselaphus* | *oby* sp. nov. | Desig. Klemann-Junior *et al*. 2025”.

**Diagnosis.** *Scaponopselaphus oby* **sp. nov.** is similar to *Scaponopselaphus diaspartos* Chatzimanolis by the coloration brown of elytra (Fig 6A) and the peg setae on the lobes of paramere of aedeagus scattered throughout its length (Figs 8E–8F). However, *Scaponopselaphus oby* **sp. nov.** can be differentiated from *Scaponopselaphus diaspartos* Chatzimanolis by the apex of the lobes of paramere of aedeagus not reaching the apex of median lobe as Figs 8A–8B (the apex of paremere of aedeagus reaching the apex of median lobe in *Scaponopselaphus diaspartos* Chatzimanolis (Figs 6A–6B in Chatzimanolis [2])). It is the unique species with one triangular tooth on middle portion of median lobe of aedeagus (Fig.?).

**Fig 6**. **Holotype of *Scaponopselaphus oby* sp. nov. (STP0358).** **A.** Dorsal habitus; **B.** Head and pronotum; **C.** Antenna.

**Measurements** males [min–max (average); N = 2]: ***Body***. BL = 11.55–11.55 (11.55), BW = 2.24–2.24 (2.24). ***Head***. HL = 1.27–1.30 (1.285), HW = 1.87–1.88 (1.875), NW = 0.99–1.00 (0.995), OL = 0.82–0.83 (0.825), OW = 0.33–0.34 (0.335), IO = 1.16–1.18 (1.17). ***Antenna***. antennomere 1 AL = 0.51–0.51 (0.51), AW = 0.18–0.18 (0.18), antennomere 2 AL = 0.29–0.29 (0.29), AW = 0.15–0.16 (0.155), antennomere 3 AL = 0.29–0.30 (0.295), AW = 0.17–0.18 (0.175), antennomere 4 AL = 0.16–0.17 (0.165), AW = 0.21–0.22 (0.215), antennomere 5 AL = 0.15–0.15 (0.15), AW = 0.22–0.22 (0.22), antennomere 6 AL = 0.16–0.16 (0.16), AW = 0.22–0.23 (0.225), antennomere 7 AL = 0.15–0.16 (0.155), AW = 0.22–0.22 (0.22), antennomere 8 AL = 0.15–0.17 (0.16), AW = 0.22–0.22 (0.22), antennomere 9 AL = 0.16–0.16 (0.16), AW = 0.21–0.22 (0.215), antennomere 10 AL = 0.16–0.17 (0.165), AW = 0.21–0.21 (0.21), antennomere 11 AL = 0.27–0.28 (0.275), AW = 0.19–0.19 (0.19). ***Thorax***. PL = 1.87–2.05 (1.96), PW = 1.87–2.05 (1.96), EL = 2.22–2.22 (2.22), EW = 2.24–2.24 (2.24), SL = 0.65–0.83 (0.74), SW = 0.79–0.79 (0.79).

**Measurements** female [N = 1]: ***Body***. BL = 10.95, BW = 2.45. ***Head***. HL = 1.21, HW = 1.75, NW = 0.99, OL = 0.79, OW = 0.32, IO = 1.11. ***Antenna***. antennomere 1 AL = 0.40, AW = 0.19, antennomere 2 AL = 0.27, AW = 0.15, antennomere 3 AL = 0.29, AW = 0.15, antennomere 4 AL = 0.13, AW = 0.20, antennomere 5 AL = 0.15, AW = 0.21, antennomere 6 AL = 0.13, AW = 0.21, antennomere 7 AL = 0.14, AW = 0.21, antennomere 8 AL = 0.14, AW = 0.20, antennomere 9 AL = 0.14, AW = 0.21, antennomere 10 AL = 0.14, AW = 0.21, antennomere 11 AL = 0.24, AW = 0.19. ***Thorax***. PL = 1.80, PW = 1.90, EL = 2.24, EW = 2.45, SL = 0.61, SW = 0.74.

**Description.** Holotype male, BL: 11.55, BW: 2.24.

**Coloration.** Dorsal head and pronotum metallic green (Figs 6A–6B). Legs including tarsi dark brown. Elytra (Fig 6A) dark brown. Abdomen (Fig 6A) dark brown, almost black, except apex of segment VII, and segments VIII–IX reddish brown. Prosternum, meso- and metaventrite from dark reddish brown to black.

**Head** (Figs 6A–6B) wider (HW: 1.88) than long (HL: 1.27) with long dark brown macrosetae along borders; dorsal surface with sparse umbilicate punctures each carrying dark brown setae, umbilicate punctures absent anteriorly in middle and denser posteriorly. Rounded hind angles, clypeus strongly emarginated, surface of vertex flat. Integument of vertex with dense microsculpture formed by fingerprint-like microlines uniformly distributed. Anterior margin of frontoclypeal region slightly emarginated (Fig 6B). Labial palpi with apical segment securiform with expanded apex (Fig 7D–7F). Eyes large and prominent (OL: 0.83; OW: 0.34), distance between eyes (IO) 1.16). Antenna (Fig 6C) with antennomeres 1–3 longer than wide, gradually club-like thickened, and without microtrichae; antennomere 1 (AL: 0.51; AW: 0.18), antennomere 2 (AL: 0.29; AW: 0.15), antennomere 3 (AL: 0.29; AW: 0.18); antennomeres 4–10 transverse and just slightly asymmetrical, antennomere 4 (AL: 0.16; AW: 0.21), antennomere 5 (AL: 0.15; AW: 0.22), antennomere 6 (AL: 0.16; AW: 0.22), antennomere 7 (AL: 0.15; AW: 0.22), antennomere 8 (AL: 0.15; AW: 0.22), antennomere 9 (AL: 0.16; AW: 0.21), antennomere 10 (AL: 0.16; AW: 0.21); antennomere 11 longer than wide (AL: 0.28; AW: 0.19); antennomeres 4–11 densely covered by microtrichae. Dorsal surface of neck with microsculpture like vertex of head and with few micropunctures (Fig 6B).

**Fig 7. Holotype of *Scaponopselaphus oby* sp. nov. (STP0358).** **A.** Abdominal sternites V–IX; **B.** Abdominal sternite VIII; **C.** Abdominal sternite IX; **D.** Head, ventral view; **E.** Labial palpomeres, anterior view; **F.** Labial palpomeres, lateral view; **G.** Mesotarsus, lateral view; H**.** Mesotarsus, ventral view.

**Pronotum** (Figs 6A–6B) wider than long (PL: 1.87; PW: 2.05) with straight anterior and lateral margins, concave and hind angles rounded. Disk with rounded umbilicate punctures, except on medial line that is abbreviated anteriorly; umbilicate punctures with dark brown setae. Integument shining, with dense microsculpture in form of rooves between umbilicate punctures. Scutellum with dense dark brown setae and micro-punctures covering surface, except its posterolateral borders.

**Elytra** (Fig 6A) wider than pronotum (EL: 2.22; EW: 2.24),; its surface polished and shiny; with denser punctuation than on pronotum with punctures almost confluent; and elytra covered by dark brown setae and, along borders with few dark brown macrosetae.

**Legs** (Fig 6A) with brown setae; protarsus dorsoventrally flattened and with spatulate pale setae ventrally; mesotarsomere 1 with spatulate pale setae ventrally (Fig 7G–7H). Mesotibia and metatibia with multiple rows of spurs on external side, and with apical spurs. Protibia without multiple rows of spurs on external side, and with single row of spurs apically.

**Abdomen** (Figs 6A and 7A) abdominal tergites III–VI with tergal basal carina; tergites III–V with subbasal curved carina. Sternite VII with round porose structure anteriorly (Fig 7A); its posterior margin of sternite VII with shallow and broad emargination at midline (Fig 7A); posterior margin of sternite VIII with deep U-shaped emargination at midline (Fig 7A–7B); sternite IX with shallow U-shaped emargination at midline (Fig 7C), it is . Tergite IX long and straight, and covered with long dark brown setae and some dark macrosetae (Fig 7A).

**Aedeagus** as in Figures 8A–8F. Paramere in parameral view (Fig 8B) divided longitudinally in two lobes slightly shorter than median lobe (Fig 8A–8B), and converging to pointed apex (Fig 8B–8E) slightly convex, and with apex with peg setae situated at middle until half of length (Fig 8E–8F). Sides of median lobe in parameral view converging to narrow pointed apex; this last with shallow median longitudinal carina and one small tooth on each side (Fig 8C–8D), and one triangular tooth on middle portion; tapering apically (Fig 8A).

**Fig 8. Aedeagus of *Scaponopselaphus oby* sp. nov., Holotype (STP0358).** **A.** Lateral view; **B.** Parameral view; **C.** Median lobe with paramere removed, parameral view; **D.** Detail of the tip of median lobe, oblique view; **E.** Detail of the tip of paramere, underside; **F.** Paramere, underside.

**Female**. Similar to male, but vertex of head is slightly convex, posterior margin of sternites VII and VIII is straight, and mesotarsomere 1 without spatulate pale setae ventrally.

**Habitat**. The specimens were collected with yellow trap in Amazon Rainforest near the area of mining activity of bauxite extraction. For a complete description of the area to see Gouvea *et al*. [23].

**Known distribution** (Fig 2). Know from municipalities of Paragominas in the state of Pará, Brazil.

**Etymology**. The epithet name “oby” is a noun in apposition taken from the native language Tupi-Guarani and means “green or blue”, due to the color of the head and pronotum.

**Remarks**. Between the male and female specimens studied, there is a variation in the metallic overtones, ranging from green to blue in the head and pronotum.

## ***Scaponopselaphus paradoxus* Asenjo, Gouvea, Valente & Klemann-Junior, sp. nov.** urn:lsid:zoobank.org:act:B2B21752-0435-456E-89BC-A8E47795DA0F

(Figs 2, 9A–C, 10A–G, 11A–F and 12)

**Type material** (6 males, 7 females)

**Holotype.** male: “PERU: LO[Department of Loreto], Ucayali, Río Pisqui, | 7°55'44.85''S / 75°33'34.66''O, | 202 m, 6-8.x[October].2011, L. Sulca”, “MUSM-ENT 0150355” (male MUSM). “HOLOTYPE [red label] | *Scaponopselaphus* | *paradoxus* sp. nov. | Desig. Klemann-Junior *et al.* 2024” (MUSM).

**Paratypes.** 5 males, 7 females:

“PERU: LO[Department of Loreto], Ucayali, | Contamana, Río Pisqui, | 7°57'22.94''S, | 75°25'8.28''W, 216 m, 1- | 3.x[October].2011, L. Sulca leg.”, “MUSM-ENT 0150349-51, 0150349-53-54” (2 males and 3 female, MUSM).

“PERU: LO[Department of Loreto], Ucayali, Río Pisqui, | 7°55'44.85''S / 75°33'34.66''O, | 202 m, 6-8.x[October].2011, L. Sulca”, “MUSM-ENT 0150356” (female, MUSM).

“PERU: LO[Department of Loreto], Ucayali, | Contamana, comunidad | nativa Roaboya, | 7°51'54.25''S, | 75°30'53.55''W, 222 m, 3- | 5.x[October].2011, L. Sulca leg.” “MUSM-ENT 0150352” (female, MUSM).

“PERÚ: CU[Department of Cuzco], La | Convención, Megantoni, | 11°41'16"S/ 73°01'12"O | 13.iii[March].2022 346 m, S. Bejar”, “MUSM-ENT 0150346” (male, MUSM).

“PERÚ: CU[Department of Cuzco], La Convención, | Echarate, San Martín Norte. | 11°45'18.8"S/72°42'26"W, | 430 m 10-14.xi[November].2010. B. | Medina y Z. Bravo.”, “MUSM-ENT 0150347” (female, MUSM).

“PERÚ: Cu[Department of Cuzco], La Convención, | Echarate, 558 m Sagari | 11°25'46.62"S/73°21'16.47"O, | 23.xi[November].2018, E. Gamboa”, “MUSM-ENT 0150348” (female, MUSM).

“PERU: CU[Department of Cuzco], La Convención, | Reserva Comunal | Matsiguenga, 581m, | 12°08'32.8”S,73°02'03.5"W, | 02.ii[February].2007, C.Castillo | Guadua-mixed forest | Pitfall trap | PM03.070302.P09”, “MUSM-ENT 0150357” (male, MUSM).

“PERU: CU[Department of Cuzco], La Convención | 12°05'7.75”S 73°03'08.06"W | 640m, 22.iv[April].2007, F. Azorsa | Bosques de colinas bajas | Yellow pan trap | PM06.070422.Y08”, “MUSM-ENT 0150358” (male, MUSM).

All paratypes with label: “PARATYPE [yellow label] | *Scaponopselaphus* | *paradoxus* sp. nov. | Desig. Klemann-Junior *et al*. 2025”.

**Diagnosis.** *Scaponopselaphus paradoxus* **sp. nov.** is similar to *Scaponopselaphus mutator* (Sharp) due to the brown color of the elytra (Fig 9A) and the peg setae concentrated near the apex of the lobes of paramere of aedeagus (Fig 10E). However, *Scaponopselaphus paradoxus* **sp. nov.** can be differentiated from *Scaponopselaphus mutator* (Sharp) by the pointed apex of the lobes of paramere of aedeagus (Fig 10E–10F) (rounded apex of the lobes of paramere in *Scaponopselaphus mutator* (Sharp) (Fig 7B–7C in Chatzimanolis [2])).

**Fig 9. Holotype of *Scaponopselaphus paradoxus* sp. nov. (MUSM-ENT 0150355).** **A.** Dorsal habitus; **B.** Head and pronotum; **C.** Antenna.

**Measurements** males [min–max (average); N = 6]: ***Body***. BL = 10.14–11.81 (10.975), BW = 2.25–2.72 (2.485). ***Head***. HL = 1.07–1.25 (1.16), HW = 1.53–1.75 (1.64), NW = 0.85–0.96 (0.905), OL = 0.68–0.82 (0.75), OW = 0.26–0.33 (0.295), IO = 0.99–1.10 (1.045). ***Antenna***. antennomere 1 AL = 0.39–0.50 (0.445), AW = 0.16–0.20 (0.18), antennomere 2 AL = 0.22–0.29 (0.255), AW = 0.12–0.15 (0.135), antennomere 3 AL = 0.22–0.27 (0.245), AW = 0.15–0.16 (0.155), antennomere 4 AL = 0.14–0.15 (0.145), AW = 0.15–0.19 (0.17), antennomere 5 AL = 0.13–0.16 (0.145), AW = 0.18–0.22 (0.20), antennomere 6 AL = 0.12–0.15 (0.135), AW = 0.18–0.22 (0.20), antennomere 7 AL = 0.12–0.16 (0.14), AW = 0.18–0.23 (0.205), antennomere 8 AL = 0.13–0.15 (0.14), AW = 0.19–0.23 (0.21), antennomere 9 AL = 0.11–0.16 (0.135), AW = 0.19–0.23 (0.21), antennomere 10 AL = 0.13–0.17 (0.15), AW = 0.20–0.24 (0.22), antennomere 11 AL = 0.26–0.30 (0.28), AW = 0.18–0.21 (0.195). ***Thorax***. PL = 1.58–1.91 (1.745), PW = 1.70–1.94 (1.82), EL = 1.91–2.41 (2.16), EW = 2.25–2.72 (2.485), SL = 0.52–0.67 (0.595), SW = 0.63–0.76 (0.665).

**Measurements** females [min–max (average); N = 7]: ***Body***. BL = 9.84–11.48 (10.66), BW = 2.46–2.76 (2.61). ***Head***. HL = 1.17–1.25 (1.21), HW = 1.65–1.77 (1.71), NW = 0.89–0.98 (0.935), OL = 0.72–0.84 (0.78), OW = 0.26–0.33 (0.295), IO = 1.05–1.11 (1.08). ***Antenna***. antennomere 1 AL = 0.45–0.50 (0.475), AW = 0.16–0.19 (0.175), antennomere 2 AL = 0.22–0.30 (0.26), AW = 0.13–0.15 (0.14), antennomere 3 AL = 0.19–0.27 (0.23), AW = 0.15–0.16 (0.155), antennomere 4 AL = 0.13–0.15 (0.14), AW = 0.17–0.19 (0.18), antennomere 5 AL = 0.13–0.14 (0.135), AW = 0.18–0.21 (0.195), antennomere 6 AL = 0.12–0.15 (0.135), AW = 0.20–0.22 (0.21), antennomere 7 AL = 0.12–0.15 (0.135), AW = 0.20–0.21 (0.205), antennomere 8 AL = 0.13–0.14 (0.135), AW = 0.21–0.22 (0.215), antennomere 9 AL = 0.12–0.15 (0.135), AW = 0.20–0.23 (0.215), antennomere 10 AL = 0.13–0.16 (0.145), AW = 0.20–0.24 (0.22), antennomere 11 AL = 0.24–0.27 (0.255), AW = 0.18–0.21 (0.195). ***Thorax***. PL = 1.72–1.91 (1.815), PW = 1.92–2.00 (1.96), EL = 2.12–2.42 (2.27), EW = 2.46–2.76 (2.61), SL = 0.61–0.71 (0.66), SW = 0.57–0.78 (0.675).

**Description.** Holotype male, BL: 10.27, BW: 2.52.

**Coloration.** Dorsal head and pronotum metallic blue with overtones green (Fig 9A–9B). Legs including tarsi light brown. Elytra (Fig 9A) dark brown. Abdomen (Fig 9A) dark brown except apex of segment VII, and segments VIII–IX light brown. Prosternum, meso- and metaventrite from dark reddish brown to black.

**Head** (Fig 9B) wider (HW: 1.71) than long (HL: 1.16), with rounded hind angles, its dorsal surface with long dark brown macrosetae along borders, and with sparse umbilicate punctures each carrying dark brown setae, umbilicate punctures absent anteriorly in middle and denser posteriorly. Integument of vertex with dense microsculpture formed by fingerprint-like microlines uniformly distributed. Clypeus strongly emarginated until its base, surface of vertex flat. Anterior margin of frontoclypeal region straight (Fig 9B). Labial palpi with apical segment securiform with expanded apex (Fig 10D–10E). Eyes large and prominent (OL: 0.78; OW: 0.32), distance between eyes (IO) 1.09. Antennomeres (Fig 9C) 1–3 longer than wide, gradually club-like thickened, and without microtrichae; antennomere 1 (AL: 0.5; AW: 0.2), antennomere 2 (AL: 0.29; AW: 0.15), antennomere 3 (AL: 0.26; AW: 0.16); antennomeres 4–10 transverse and just slightly asymmetrical, antennomere 4 (AL: 0.15; AW: 0.19), antennomere 5 (AL: 0.16; AW: 0.22), antennomere 6 (AL: 0.15; AW: 0.22), antennomere 7 (AL: 0.16; AW: 0.23), antennomere 8 (AL: 0.15; AW: 0.23), antennomere 9 (AL: 0.16; AW: 0.23), antennomere 10 (AL: 0.17; AW: 0.22); antennomere 11 longer than wide (AL: 0.29; AW: 0.2); antennomeres 4–11 densely covered by microtrichae. Dorsal surface of neck with microsculpture like vertex of head and with few micropunctures (Fig 9B).

**Pronotum** (Fig 9A–9B) wider than long (PL: 1.83; PW: 1.94), with anterior margin almost straight, lateral margin concave and hind lateral angles and posterior margin rounded. Disk with rounded umbilicate punctures, except on medial line that is abbreviated anteriorly; umbilicate punctures with light brown setae. Integument shining, with dense microsculpture in form of rooves between umbilicate punctures. Scutellum with dense dark brown setae and micropunctures covering its surface.

**Elytra** (Fig 9A) wider than pronotum (EL: 2.24; EW: 2.52); surface polished and shiny; denser punctuate than on pronotum; punctures almost confluent; covered with dark brown setae and, along borders with few dark brown macrosetae.

**Leg** (Fig 9A) with brown setae; protarsus dorsoventrally flattened and with spatulate pale setae ventrally; mesotarsomere 1 with spatulate pale setae ventrally (Fig 10F–10G). Mesotibia and metatibia with multiple rows of spurs on external side, and with apical spurs. Protibia without multiple rows of spurs on external side, and with single row of spurs apically.

**Abdomen** (Fig 9A) abdominal tergites III–VI with tergal basal carina; tergites III–V with subbasal curved carina. Sternite VII with round porose structure anteriorly (Fig 10A); its posterior margin of sternite VII with shallow and broad emargination at midline (Fig 10A); posterior margin of sternite VIII with deep U-shaped emargination at midline (Fig 10B); sternite IX with profound U-shaped emargination at midline (Fig 10C), it is long and straight covered with long dark brown setae and some dark macrosetae.

**Fig 10. Holotype of *Scaponopselaphus paradoxus* sp. nov. (MUSM-ENT 0150355).** **A.** Abdominal sternites V–IX; **B.** Abdominal sternite VIII; **C.** Abdominal sternite IX; **D.** Head, ventral view; **E.** Labial palpomeres, lateral view; **F.** Mesotarsus, lateral view; **G.** Mesotarsus, ventral view.

**Aedeagus** as in Figures 11A–11F. Paramere in parameral view (Fig 11B and 11F) divided longitudinally in two lobes narrower and shorter than median lobe (Fig 11B), and converging to pointed apex (Fig 11E), wich is slightly convex (Fig 11A), and with peg setae concentrated on apex of lobes (Fig 11E–11F). Sides of median lobe converging to narrow pointed apex (Fig 11B and 11C); apical region with one small tooth on each connected by one carina, resembling to semicircle (Fig 11C–11D); median lobe tapering apically (Fig 11B–11C).

**Fig 11. Aedeagus of *Scaponopselaphus paradoxus* sp. nov. Holotype (MUSM-ENT 0150355).** **A.** Lateral view; **B.** Parameral view; **C.** Median lobe with paramere removed, parameral view; **D.** Detail of the tip of median lobe, oblique view; **E.** Detail of the tip of paramere, underside; **F.** Paramere, underside.

**Female**. Similar to male, but vertex of head slightly convex, posterior margin of sternites VII and VIII straight, and mesotarsomere 1 without spatulate pale setae ventrally.

**Habitat**. Some specimens were collected with yellow trap and pitfall trap without bait in *Guadua*-mixed forest and low hill forest respectively from Cuzco.

**Known distribution** (Fig 2). Known from localities of Río Pisqui, Comunidad nativa Roaboya in the department of Loreto, and localities of Megantoni, San Martin Norte Sagari and Reserva Comunal Matsiguenga in the department of Cuzco, Peru.

**Etymology**. The specific name “*paradoxus”* is a noun in apposition.

**Remarks**. Between the male and female specimens studied, there is a variation in the metallic overtones, ranging from green to blue and purple-blue in the head and pronotum.

## **Additional material examined**

***Scaponopselaphus mutator*** (Sharp, 1876)

**French Guiana:** Itoupé, DZE 570, 03°01'23"N, 53°05'44"W, 800m, 17.iii.2010, *leg.* SEAG (1 male, UEA).

**Note:** Itoupé is a new locality record for French Guiana (Fig 2). The species was previously known from Brazil, Peru, Suriname and French Guiana [2]

## **Key to species of *Scaponopselaphus* Scheerpeltz**

Key to species is provided in DELTA version in S1 Key and pictorical key (Fig 12).

1. Elytra black (Fig 3A) ……………………………. ***Scaponopselaphus caribi* n. sp.**

- Elytra from light brown to dark brown (Figs 6A and 9A) …………………………… **2**

2. Peg setae scattered throughout the length of the lobes of paramere of aedeagus (Fig 8E–8F)………………………………………………………………….…………...…. **3**

- Peg setae concentrated near the apex of the lobes of paramere of aedeagus (Fig 11E–11F) ……………………………………………………………………………………..**4**

3. Apex of paramere of aedeagus not reaching the apex of median lobe (Fig 8A–8B); one small tooth on the middle portion of the median lobe (Fig 8C–8D) … ***Scaponopselaphus oby* n. sp.**

- Apex of paramere of aedeagus reaching the apex of median lobe (Fig 6A–6B in Chatzimanolis [2]); small tooth on the middle portion of the median lobe lacking… ***Scaponopselaphus diaspartos* Chatzimanolis**

4. Apex of the lobes of paramere of aedeagus rounded (Fig 7B–7C in Chatzimanolis [2]) … ***Scaponopselaphus mutator* (Sharp)**

- Apex of the lobes of paramere of aedeagus pointed (Fig 11E–11F) … ***Scaponopselaphus paradoxus*** **n. sp.**

**Fig 12.** **Pictorial Key to species of *Scaponopselaphus* Scheerpeltz.**

## **Ecological niche modeling of genera *Scaponopselaphus* for South America**

A total of 25 independent occurrences were recorded. Principal Component Analysis (PCA) identified 19 components (table in S2 table), with the first six accounting for 96.9% of the total variability, and the first two components 74.9%. The first six axes were retained as the new predictor variables. For the first component, the variables with the highest eigenvectors were BIO6 (0.292) and BIO11 (0.284), with none standing out prominently. For the second component, the variables with the highest eigenvectors were BIO14 (0.400) and BIO18 (0.399).

The ensemble model obtained excellent metrics (Table 1): AUC = 0.97 ± 0.05, TSS = 0.89 ± 0.13, and Jaccard = 0.90 ± 0.10. The algorithms also showed good metrics individually, with Random Forest performing better (AUC = 0.97 ± 0.02, TSS= 0.91 ± 0.07, Jaccard= 0.91 ± 0.07). GLM performed comparatively less well than the other algorithms (AUC = 0.88 ± 0.15, TSS = 0.71 ± 0.32, and Jaccard = 0.80 ± 0.18).

**Table 1. Evaluation table with metrics of ecological niche modeling.**

| **Algorithm** | **AUC** | **TSS** | **Jaccard** |
| --- | --- | --- | --- |
| **Mean Ensemble Algorithm - MEA** | 0.97 ± 0.05 | 0.89 ± 0.13 | 0.90 ± 0.10 |
| **Maxent Defauld - MXD** | 0.96 ± 0.05 | 0.87 ± 0.11 | 0.89 ± 0.10 |
| **Random Forest - RDF** | 0.97 ± 0.02 | 0.91 ± 0.07 | 0.91 ± 0.07 |
| **Support Vector Machine - SVM** | 0.95 ± 0.06 | 0.85 ± 0.15 | 0.88 ± 0.11 |
| **Generalized Linear Model - GLM** | 0.88 ± 0.15 | 0.71 ± 0.32 | 0.80 ± 0.18 |

Algorithms such as MXD, SVM and RDF restrict the most suitable areas mainly to the Amazon region (Fig 13). Although MXD has a similar range to SVM and RDF, the boundaries between more and less suitable areas are not as well defined as both. GLM has a wide coverage that extends to portions of Argentina and Chile, but it was the algorithm that obtained the lowest metrics and the highest standard deviation. To deal with the uncertainty of the different algorithms used, the Ensemble model provides a more robust and balanced projection based on the average of each algorithm.

**Fig 13.** **Comparison of distribution models for *Scaponopselaphus* Scheerpeltz.**

The areas predicted by the ensemble model (Fig 14) to be most suitable are concentrated in northern South America, particularly in the central region of the Amazon biome. In Brazil, areas of high suitability were identified in the states of Acre, Amazonas, Pará, Roraima, and Amapá. In Colombia and Venezuela, the most suitable areas are located in the southern parts of both countries. In Peru and Ecuador, suitable areas include not only the Amazon tropical forest, but also montane ecosystems along the slopes of the Andes.

**Fig 14. Ensemble ecological niche model of *Scaponopselaphus* Scheerpeltz.**

## **Biological notes**

The species of *Scaponopselaphus* were collected in evergreen Tropical Forests, *Guadua*-mixed forests, collected with Winkler, Pitfall trap, Moerick trap, Pink pan trap or flight intercept traps. Until now, nothing is known about his food preferences.

# **Discussion**

# **Taxonomy**

The new species belong to the genus *Scaponopselaphus*, based on the presence of the following characters used by Chatzimanolis [2]: head with distinctive microsculpture (Figs 3B, 6B and 9B); labial palpomere 3 securiform (Figs 4D–4E, 7D–7F and 10D–10E); pronotum with broad and convex lateral margins (Figs 3A–3B, 6A–6B and 9A–9B); mesotarsomere 1 in males with spatulate setae (Figs 4F–4G, 7G–7H and 10F–10G); tergites III–V with curved carina (Figs 3A, 6A and 9A), and sternite VII in males with porose structure (Figs 4A, 7A and 10A).

# **Distribution and biological notes**

The ecological niche of *Scaponopselaphus* Scheerpeltz has been modeled for the first time, with high performance, demonstrating the reliability of the algorithm in predicting suitable regions for the genus. Our study identifies potential areas across the Amazon region, corresponding to existing knowledge gaps regarding the genus distribution. However, it is important to emphasize that the predicted distribution does not necessarily reflect the actual distribution of the taxon, since the model does not account for essential factors influencing the realized distribution, such as dispersal capabilities, geographic barriers, and ecological interactions. However, these predictions provide valuable insights, guiding future research aimed at addressing taxonomic and distributional gaps. They enable targeted collection efforts in regions identified as highly suitable. Currently, biological information on *Scaponopselaphus* Scheerpeltz is also limited, likely due to factors such as observational challenges, a lack of focused studies on the genus, and the rarity of its specimens in scientific collections. This lack of knowledge hinders the ability to conduct targeted sampling, as there are no records of the specific environments occupied. Some inferences, however, can be drawn from the genus morphological traits. The morphology among species is highly conserved, with differences observed almost exclusively in male genitalia, which suggests that species within the genus occupy similar ecological niches. Other inferences can be drawn from the field sample data, since the majority of the species of *Scaponopselaphus* collected have been obtained from intercepted flights traps of tropical forests, which highlights the effectiveness in sampling these species with these methods, however, there is still doubts about the habitat occupied since flight interception traps may collect taxa from a variety of environments such as canopy and understory. Further targeted efforts exploring target regions could help knowledge gaps in taxonomy, distribution and biology, providing a better understanding of the genus and its ecological requirements. Such research will be crucial for guiding future conservation and ecological studies.

# **Acknowledgments**

We would like to thank Conselho Nacional de Desenvolvimento Científico e Tecnológico (CNPQ) and Coordenação de Aperfeiçoamento de Pessoal de Nível Superior (CAPES) for the constant financial support granted to LKJ, and Precious Woods – Mil Madeiras Preciosas for the logistical support that made it possible to capture specimens of the species described here. We appreciate the Laboratório de Invertebrados (LA-INV) assistance and support of our colleagues, with special acknowledgment to Bruna Façanha for her support and patience throughout the process. The participation of AA was funded by CONCYTEC through the PROCIENCIA program within the framework of the call "Interinstitutional Alliances for Doctorate Programs" according to contract PE501084299-2023-PROCIENCIA-BM.

# **References**

1. Guayasamin JM, Ribas CC, Carnaval AC, Carrillo JD, Hoorn C, Lohmann LG, et al. 2021. Chapter 2: Evolution of Amazonian Biodiversity. In: Nobre C, Encalada A, Anderson E, Roca Alcazar FH, Bustamante M, Mena C, et al. Editors. Amazon Assessment Report 2021. United Nations Sustainable Development Solutions Network, New York, USA.

2. Chatzimanolis S. A review of the genus *Scaponopselaphus* Scheerpeltz (Insecta: Coleoptera: Staphylinidae). Biodivers Data J. 2015;3: e4735. doi: 10.3897/BDJ.3.e4735

3. Asenjo A, Irmler U, Klimaszewski J, Chandler DS, Fierros-López HE, Vieira JS. Staphylinidae (Insecta: Coleoptera) in Latin America: synopsis, annotated catalog, diversity and distribution. Zootaxa. 2019;4621(1): 1–406. doi: 10.11646/zootaxa.4621.1.1

4. Guisan A, Zimmermann NE. Predictive habitat distribution models in ecology. Ecol Modell. 2000;135: 147–186.

5. Naomi SI. Comparative morphology of the Staphylinidae and the allied groups (Coleoptera, Staphylinoidea) II. Cranial structure and tentorium. Kontyû. 1988;55(4): 666–675.

6. Naomi SI. Comparative morphology of the Staphylinidae and the allied groups (Coleoptera, Staphylinoidea). X. Eighth to 10th segments of abdomen*.* Japanese Journal of Entomology*.* 1989;57(4): 720–733.

7. GBIF.org (05 November 2024) GBIF Occurrence Download of *Scaponopselaphus.* In: <https://doi.org/10.15468/dl.pvs4zy>

8. QGIS.org (2024). QGIS Geographic Information System. Open Source Geospatial Foundation Project. In: [http://qgis.org](http://qgis.org/)

9. Fick SE, Hijmans RJ. WorldClim 2: new 1km spatial resolution climate surfaces for global land areas. Int. J. Climatol. 2017;37(12): 4302–4315.

10. De Marco P, Nóbrega CC. Evaluating collinearity effects on species distribution models: An approach based on virtual species simulation. PLoS ONE. 2018; 13(9): e0202403. doi: 10.1371/journal.pone.0202403

11. Phillips SJ, Anderson RP, Schapire RE. Maximum entropy modeling of species geographic distributions. Ecol. Modell. 2006;190: 231–259. doi: 10.1016/j.ecolm odel.2005.03.026

12. Phillips SJ, Anderson RP, Dudík M, Schapire RE, Blair ME. Opening the black box: An open-source release of Maxent. Ecography. 2017;40: 887–893. doi: 10.1111/ecog.03049

13. Karatzoglou A, Smola A, Hornik K, Zeileis, A. Kernlab– an S4 package for kernel methods in R. J. Stat. Softw. 2004;11(9): 1–20. doi: 10.18637/jss.v011.i09

14. R Core Team (2018) R: a language and environment for statistical computing R foundation for statistical computing. Vienna, Austria 2018

15. Liaw A, Wiener M. Classification and regression by Ran-dom Forests. R News. 2002;2/3: 18–22. [online] URL http://CRAN.R-project.org/doc/Rnews/.

16. Fielding AH, Bell JF. A review of methods for the assessment of prediction errors in conservation presence/absence models. Environ. Conserv. 1997;24: 38–49.

17. Leroy B, Delsol R, Hugueny B, Meynard CN, Barhoumi C, Barbet-Massin M, Bellard C. Without quality presence–absence data, discrimination metrics such as TSS can be misleading measures of model performance. J. Biogeogr. 2018;45: 1994–2002. doi: 10.1111/jbi.13402

18. Allouche O, Tsoar A, Kadmon, R. Assessing the accuracy of species distribution models: prevalence, kappa and the true skill statistic (TSS). J. Appl. Ecol. 2006; 43: 1223-1232. doi: 10.1111/j.1365-2664.2006.01214.x

19. Andrade AFA de, Velazco SJE, Júnior PDM. ENMTML: An R package for a straightforward construction of complex ecological niche models. Environ. Model. Softw. 2020;125: e104615. doi: 10.1016/j.envsoft.2019.104615

20. R Core Team (2024) R: a language and environment for statistical computing R foundation for statistical computing. Vienna, Austria 2024

21. Moura RS, Noriega JA, Cerboncini RAS, Vaz-De-Mello F, Klemann-Junior L. Dung beetles in a tight-spot, but not so much: Quick recovery of dung beetles assemblages after low-impact selective logging in Central Brazilian Amazon. For Ecol Manage. 2021;494: e119301. doi: 10.1016/j.foreco.2021.119301

22.Veloso HP, Range-Filho ALR, Lima JCA. Classificação da vegetação brasileira, adaptada a um sistema universal. 1st ed. Rio de Janeiro: Instituto Brasileiro de Geografia e Estatística (IBGE); 1991.

23. Gouvea B, Valente RM, Asenjo A. A new species from the eastern Amazon rainforest with lectotype designations and key for Brazilian *Oxarthrius* Reitter, 1883 (Coleoptera: Staphylinidae: Pselaphinae). Zootaxa. 2023;5380(2): 134–150. doi: 10.11646/zootaxa.5380.2.2

# **Supporting information**

**S1 Table. Measurements of specimens of *Scaponopselaphus* *caribi* n. sp., *Scaponopselaphus oby* n. sp. and *Scaponopselaphus* *paradoxus* n. sp., comparing the holotype specimen with the paratypes.**

(XLSX)

**S2 Table. Principal component analysis (PCA) of 19 climate variables, considering the occurrence records of Scaponopselaphus Scheerpeltz**

(XLSX)

**S1 File. File with all data for ecological niche modeling.**

(ZIP)

**S1 Key. key to species of the genus *Scaponopselaphus* Scheerpeltz in DELTA (DEscription Language for TAxonomy) version.**

(DLT)

**S1 Map. Distribution map of the species of *Scaponopselaphus* Scheerpeltz in the KMZ version for Google Earth.**

(KMZ)
